# Techno-economic assessment of green hydrogen production integrated with hybrid and organic Rankine cycle (ORC) systems

**DOI:** 10.1016/j.heliyon.2024.e25742

**Published:** 2024-02-08

**Authors:** Suresh Baral, Juraj Šebo

**Affiliations:** a Faculty of Science and Technology, School of Engineering, Pokhara University, 33700, Kaski, Nepal; b Faculty of Mechanical Engineering, Technical University of Košice, Letná 9, 04200, Košice, Slovakia

**Keywords:** Green hydrogen, Hybrid systems, Hydrogen production cost, Techno-economic, ORC systems

## Abstract

This study aims to determine the most cost-effective approach for production of green hydrogen, a crucial element for global decarbonization efforts despite its high production costs. The primary research question addresses the optimal and economically viable strategy for green hydrogen production considering various scenarios and technologies. Through a comprehensive analysis of eight scenarios, the study employs economic parameters such as net present value, minimum production cost, payback period and sensitivity analysis. The analysis is validated using established economic metrics and real-world considerations to ensure feasibility. The results suggest that a hybrid system combining solar photovoltaic (PV) with storage and onshore wind turbines is a promising approach yielding a minimum cost of $3.01 per kg of green hydrogen, an internal rate of return (IRR) of 5.04% and 8-year payback period. These findings provide a practical solution for cost-effective green hydrogen production supporting the transition to sustainable energy sources. The study also highlights the future potential of integrating solar thermal (CSP) with an organic Rankine cycle (ORC) system for waste heat recovery in hydrogen production. The sensitivity analysis provides the importance of capacity factor, levelized cost of hydrogen, capital expenditure and discount rate in influencing production costs.

## 1 Introduction

Green hydrogen is emerging as a critical component of the world's future energy mix, with the potential to decarbonize various industries and contribute to achieving the climate goals of many countries. Hydrogen is a versatile energy carrier that can be used in a variety of applications, such as fuel cells for transportation and power generation, energy storage, and industrial processes [1]. However, the current production of hydrogen is primarily based on fossil fuels, resulting in significant greenhouse gas emissions. Green hydrogen production using renewable energy sources, such as solar, hydro, geothermal and wind have the potential to provide a sustainable and clean energy solution. As for the future cost of green hydrogen, there are several factors that could impact it, such as advancements in technology, economies of scale, and government policies and incentives. It is predicted that the cost of green hydrogen could decline significantly in the coming years, making it more competitive with traditional fossil fuels [2]. A report published by the International Renewable Energy Agency (IRENA) suggests that the cost of green hydrogen could fall by 50% or more by 2030, driven by declining costs for renewables and electrolyzers, as well as improvements in efficiency and economies of scale [3].

The techno-economic analysis of green hydrogen production is crucial for evaluating the feasibility of large-scale production and deployment. Several articles have discussed the techno-economic analysis of green hydrogen, considering various factors such as the cost of production, energy efficiency, and the potential for renewable energy integration [[4], [5], [6]].

While the literature presents numerous studies on the techno-economic analysis of green hydrogen production using various renewable energy sources, there is a notable gap in research regarding the integration of multiple renewable energy sources, particularly organic Rankine cycle (ORC) systems, for green hydrogen production. The existing studies tend to focus on individual renewable sources or specific integration scenarios, but there is a lack of comprehensive analysis that evaluates the feasibility of a hybrid system combining solar PV, wind, hydropower, geothermal and ORC technology. Additionally, few studies have addressed the economic viability of such hybrid systems for producing green hydrogen. To the best of our knowledge, there are rare studies which explore the economic viability of producing green hydrogen through the integration of multiple renewable energy sources and ORC systems. In addition, the findings of this study may provide significant advantages to countries that aim to develop and implement national policies regarding hydrogen strategies. The presented results can be useful in forecasting the future adoption of hydrogen as a primary fuel source. The primary research question addressed by this study is: What is the techno-economic feasibility of producing green hydrogen through the integration of a hybrid system combining solar PV, wind turbines, hydropower plants, geothermal and an organic Rankine cycle (ORC) systems, along with comparison by creating several scenarios?

The primary aim of this study is to evaluate the techno-economic feasibility and conduct a comparative analysis of different scenarios for producing green hydrogen. This assessment involves the use of performance metrics, including net present value (NPV), minimum production cost, payback period and sensitivity analysis to thoroughly evaluate and compare a range of integration scenarios.

## 2 Literature review

Various studies have been conducted on the techno-economic analysis of green hydrogen production, with each study proposing different methods to increase the efficiency of green hydrogen production while minimizing its cost by using solar energy. For instance, Fang et al. [7] proposed a combined system that utilizes fossil fuels and solar energy for generating hydrogen, achieving a solar to hydrogen efficiency of 37.47% with CO_2_ reduction in an approximate value of 15 kg/kgH_2_. On the other hand, Chatterjee et al. [8] reviewed PV/photo-electrocatalysis for green hydrogen production, with a focus on the major challenges and advantages in PV-PEM, as well as the design of materials for affordable production. The study showed that PV-thermal integration system could reduce the payback period due to maximum solar energy utilization.

Similarly, Mastropasqua et al. [9] revealed that affordable solar hydrogen generation requires pressuring and high current density operation. Their economic analysis shows that the levelized cost of hydrogen is 5.9 €/kg, concluding that it is not competitive in the present scenario with steam reforming hydrogen production. Monnerie et al. [10] studied CSP that uses a solar tower for hydrogen production with high-temperature electrolyzer, focusing on high thermal storage time period. The solar to hydrogen efficiency achieved was 14% greater when compared with two scenarios, namely mobility use and industrial use, with thermal storage of 12 h.

Laoun et al. [11] developed the model for solar PV-PEM electrolysis for hydrogen generation with multi-objective optimization to minimize the loss in energy conversion. The study concluded that the developed model accurately determines the performance of the solar PV-PEM system that helps for decision making in investment. The study by Karayel et al. [12] investigated the solar-based hydrogen production potential in Turkey, estimating the potential for green hydrogen production to be between 415 and 427 million tons. Abbas et al. [13] evaluated the economic and technical feasibility of using a 22 kWp photovoltaic energy system for hydrogen production and indicated that it is a viable and promising technique with an annual production rate of 1891.12 kg/year and a levelized cost of $3.79/kg in Iraq.

Moreover, Hassan et al. [14] assessed the feasibility of constructing a 20 MW solar photovoltaic power plant for electricity generation in four main cities in Iraq, considering factors such as solar irradiance, wind speed, and ambient temperature. The results showed the most influencing parameter for hydrogen generation is solar irradiance when observed in the mid-western region and the greatest influence of ambient temperature in the southern regions.

Other studies on the techno-economic analysis of green hydrogen production by using wind energy sources are discussed. Ahshan et al. [15] conducted an economic analysis of producing green hydrogen in Oman using wind power, and found that the levelized cost of hydrogen (LCOH) ranges from 3.37 to 6.13 USD/kg. Other studies on the techno-economic analysis of green hydrogen production by using wind energy sources are discussed.

Mohsin et al. [16] evaluated the feasibility of hydrogen production from wind power in Pakistan, with the cost of electricity ranging from $0.0844 to $0.0864 per kWh, and the supply cost of renewable hydrogen ranging from $5.30 to $5.80 per kg-H_2_. Haiyang et al. [17] analyzed the data from China's Western Inner Mongolia to explore the potential of using wind power for renewable hydrogen production as a cost-effective and eco-friendly alternative to the coal-dominated hydrogen manufacturing system.

Ayodele and Munda [18] investigated the production of hydrogen from wind energy at 15 different sites in five major provinces in South Africa, identifying site S5 to have the highest hydrogen production potential, and WT9 to have the lowest cost of electricity generation. Mostafaeipour et al. [19] evaluated hydrogen production by wind energy for agricultural and industrial sectors in four cities in Iran, finding that the production of hydrogen was 5253 kg annually in Ardebil city.

Rezaei et al. [20] investigated the techno-economic analysis of co-production of electricity and hydrogen from wind, with levelized cost of hydrogen ranging from 1.375 to 1.59 $/kg, payback period from 3.91 to 8.41 years, and rate of return from 9.87 to 21.55%. Jovan et al. [21] examined the potential for green hydrogen production in a run-of-river hydropower plant, where a control algorithm was presented to regulate the amount of hydrogen production, and the economic viability of using hydrogen for local public transport was discussed. Jovan et al. [22] conducted a case study on HPP, finding that the cost of producing green hydrogen is 1.36 €/kg if excess electricity is used, or 3.86 €/kg if electricity can be sold for 50 €/MWh on the free market.

Similarly the literature on production of hydrogen by hydropower plant has been discussed here. Thapa et al. [23] analyzed the possibility of producing green hydrogen from hydropower energy in Nepal and using it in the transportation sector, with the potential for hydrogen production varying between 63,072 and 3,153,360 tons in 2030. Andrus et al. [24] explored the feasibility of generating green hydrogen from surplus hydropower energy in the Columbia River System in the United States, with the potential to produce 2.22 to 8.96 million kilograms of H_2_ on a monthly average basis. Zwickl-Bernhard and Hans [25] conducted a study on the profitability of green hydrogen production using hydropower electricity generation for transportation. They developed a bi-level optimization framework between a hydropower plant owner and a transportation firm and found that profitable green hydrogen production requires a CO_2_ price above €245 per ton.

Besides, Sijan et al. [26] conducted simulations for the production of hydrogen and ammonia to be used for urea production. Their findings showed that the levelized cost of hydrogen (LCOH) and the levelized cost of ammonia (LCOA) were $1582/ton and $314/ton respectively. Moreover, the study suggests that this method of production could be a promising option for Nepal, as the electricity required for the process could be generated from hydropower sources. According to the assessment conducted by Gyanwali et al. [27], the integration of hydropower and hydrogen in a grid system can facilitate energy growth. The study suggests that to achieve complete decarbonization of the transport sector by 2050, an additional 14 GW of energy will be required for the implementation of grid-connected hydrogen storage systems and charging stations for both electric and hydrogen-powered vehicles in Nepal.

On the other hand, there are several articles discussing the potential of geothermal energy for hydrogen production. Study by the authors,Kanoglu, Ali and Ceyhun [28] developed thermodynamic models for different scenarios, showing that higher geothermal temperatures lead to increased hydrogen production. In one scenario, 1 kg of 200 °C geothermal water could produce 1.34 g of hydrogen through reversible operation.

AlZaharani, Dincer, and Naterer [29] evaluated a geothermal energy-based integrated system for power and hydrogen production. They used a combined power plant with a supercritical CO_2_ Rankine cycle and an ORC cascade, along with a heat recovery system for domestic hot water. The system's energy and exergy efficiencies were 13.67% and 32.27%, respectively, and it generated a total net power of 18.59 MW, which can produce 245 kg/h of hydrogen.

Wang et al. [30] proposed a high-efficiency power and hydrogen co-production framework using renewable resources such as biomass and geothermal energy. The system consisted mainly of a gas turbine and a geothermal-assisted Rankine unit, which extracted waste heat to power a water electrolyzer for hydrogen production. The optimized cogeneration framework operated with an exergetic efficiency of 42.37% and a levelized product cost of 68.52 $/MWh, emitting 0.7443 kg/kWh CO_2_, leading to performance enhancements by 7.5%, 9.0%, and 7.7% compared to the basic design. Finally, Hashemian and Noorpoor [31] proposed a geothermal-biomass-powered multi-generation plant to produce electricity, heating, cooling, hydrogen, and freshwater. They optimized the plant using a multi-criteria approach and evaluated its environmental impact based on NO_x_ and CO_2_ emissions and found that hydrogen production of 88.12 kg/h along with 39.85 MW and 126.36 MW of heating and cooling capacity. Solar and wind power energy sources are known for their variability which poses a challenge for ensuring a reliable and secure energy supply. To overcome this challenge, one possible solution is to combine hydrogen production with hybrid systems. However, the high cost of hydrogen production systems, particularly the electrolyzer, must be addressed to make this feasible. To address this challenge, various integration schemes have been proposed to reduce the cost of green hydrogen production, including the integration of green hydrogen production with renewable energy sources, energy storage systems, and other industrial processes. Hybrid systems that combine multiple energy sources and conversion technologies have also been explored as a way to improve the efficiency and cost-effectiveness of green hydrogen production. The main aim of the study is on a specific approach to this challenge, which is to integrate an Organic Rankine Cycle (ORC) system to produce green hydrogen, and assesses its technical and economic viability. Organic Rankine Cycle technology, which is a type of power generation technology that uses organic fluids, such as hydrofluorocarbons (HFCs) or hydrocarbons (HCs), as working fluids instead of water or steam [32,33]. ORC technology can be used for hydrogen production as it allows the recovery of waste heat from various sources, such as concentrated solar power (CSP), geothermal energy or industrial processes, which can be used to power a water electrolyzer for hydrogen production.

## 3 Methodology

**Fig. 1:**
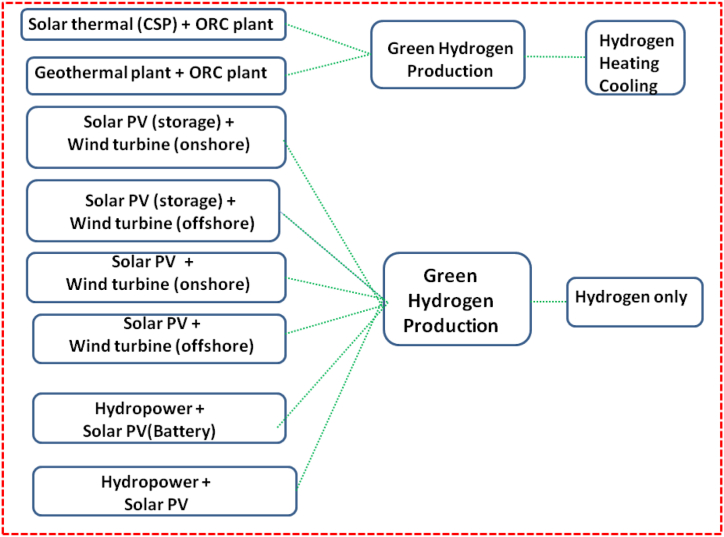
Scenarios for hybrid systems for hydrogen production.

The objective of the article is to evaluate the techno-economic feasibility of producing green hydrogen under eight distinct scenarios. These scenarios involve a range of renewable energy (RE) systems, with two scenarios incorporating the integration of ORC systems. The layout of the proposed integration system is illustrated in Fig. 1 and is the different pathways and scenarios considered for green hydrogen production in this study.

### 3.1 Economic analysis for green hydrogen production

The development of multiple scenarios stems from the varying potential of renewable energy (RE) sources across different regions of the world. By combining these hybrid systems, countries can efficiently produce hydrogen to meet their specific needs. Moreover, in situations of excess electricity production, hybrid systems can also be utilized effectively. Here are the main assumptions for conducting of economic analysis.

1. In developing these scenarios, a specific cost per kW size has been selected are compared for each hybrid systems.
2. The hybrid plants were operated for based on capacity factors of the systems. The capacity factors with minimum and maximum values are taken for estimation.
3. The hybrid systems lifetime is estimated to be 25 years expect hydropower plant which is 30 years. It is assumed that during the initial period (first year) there is no any generation of revenue since it is constructional phase of the systems.
4. The operational cost is assumed to be 2% annually to that of capital cost and the discount rate is assumed to be 5%.
5. No additional cost such as replacement of batteries, hydrogen compressor and control equipment are considered.

It is crucial to have an understanding of the capacity factors of different technologies involved in hybrid renewable systems to accurately assess their power production. Table 1 provides an overview of the costs associated with each renewable energy source for producing hydrogen with varying capacity factors, where higher capacity factors result in more efficient and productive energy systems. Solar, wind, and hydropower have different capacity factors, which are influenced by factors such as location and weather conditions. Geothermal energy sources have the highest capacity factor, while the ORC system has the lowest capacity factor, as indicated in the table. The economic feasibility of green hydrogen production is largely determined by the cost of generating electricity from renewable resources in a specific location.

**Table 1:** Categorization of CAPEX for integrated systems with capacity factor.

Renewable Sources	CAPEX ($/kW)	Capacity factor (%)	Lifetime (Years)	Reference
Solar energy				
CSP (without TES)	6476	30–50	25	[[34], [35], [36]]
PV systems (Without battery storage)	883	10–25	25	[9,37,38]
PV systems (With battery storage)	1207	20–50	25	[34,38,39]
**Wind Turbine**				
Wind Turbine (Offshore)	3720	40–60	25	[38,40,41]
Wind Turbine (Onshore)	1370	25–45	25	[38,40,41]
**Hydroelectric plant**	2528	30–50	30	[21,35]
**Geothermal plant**	6629	70–90	25	[35,42]
Electrolyzer (Alkaline)	930	30–50	25	[38,40]
Compressor	1860	–	10	[38]
ORC system	3500	8–11	25	[32]
Hydrogen tank ($/kg)	350		25	[43]

For determining the cost of hydrogen production, first of all annual equivalent cost (AEC) should be known for each component. The annual equivalent cost (AEC) is a method used in engineering economics to calculate the annual cost of owning, operating, and maintaining an asset over its expected lifespan. This can be calculated by considering the total capital expenditures (CAPEX), operating expenditures (OPEX) and revenue generation from the hybrid system during the plant life period [44,45].

(i) $$AEC=-CAPEX+\sum_{n=1}^{N}\frac{{\text{Revenue}}_{n}\times i}{1-{\left(1+i\right)}^{-n}}$$

here are the governing equations for some of the key economic metrics used in this analysis.

The Net Present Value (NPV) of a hybrid system over its lifetime and is given by following equation [46]:

(ii) $$NPV=-CAPEX+\sum_{n=1}^{N}\frac{{\text{Revenue}}_{n}-{\text{OPEX}}_{n}}{{\left(1+i\right)}^{n}}$$

Levelized cost of hydrogen production (LCOH) is calculated by dividing the total cost by the total hydrogen production and is given by following equation [44,46]:

(iii) $$LCOH=\frac{CAPEX+\sum_{n=1}^{N}\frac{{\text{OPEX}}_{n}}{{\left(1+i\right)}^{n}}}{\sum_{n=1}^{N}\frac{{\text{AEC}}_{n}}{{\left(1+i\right)}^{n}}}$$

The internal rate of return (IRR) is the interest rate value that causes the NPV to be zero. If the internal rate of return is higher than the interest rate, the investment is considered worthwhile. In other words, Internal Rate of Return (IRR) represents the rate at which the present value of the cash inflows equals the present value of the cash outflows of a hybrid system and is given by following equation [44,45]:

(iv) $$NPV=\sum_{n=0}^{N}\frac{{CF}_{n}}{{\left(1+IRR\right)}^{n}}=0$$

Profitability index (PI): The profitability index represents the ratio of the present value of the benefits (revenue) to the present value of the costs of a hybrid system and is given by Refs. [32,45]:

(v) $$PI=\frac{{Revenue}_{n}}{{OPEX}_{n}}$$

Payback Period (PB): The payback period represents the time it takes for a hybrid system to recover its initial CAPEX cost through cash inflows (revenue) and is given by Ref. [46]:

(vi) $$PB=\frac{CAPEX}{Annual\;revenue}$$

At present, the production of green hydrogen utilizing electrolyzer systems and renewable electricity is anticipated to be costlier than traditional techniques. Despite recent technological advancements that have led to a reduction in the expenses of critical materials for electrolyzers, the whole system still lacks profitability. Consequently, it is crucial to perform comparative economic analyses of various water electrolysis technologies to determine ways to decrease the production cost of green hydrogen. Currently there are four types of electrolyzers such as Alkaline, Polymer Electrolyte Membrane (PEM), Solid Oxide Electrolyzer Cell (SOEC) and Anion Exchange Membrane (AEM). Alkaline Electrolyzer electrolyzer uses an alkaline solution as an electrolyte to produce hydrogen and oxygen through the process of water electrolysis where as Polymer Electrolyte Membrane (PEM) is a type of proton-conducting membrane commonly used in fuel cells and electrolyzers, enabling the conduction of ions for efficient operation and finally Solid Oxide Electrolyzer Cell (SOEC) is an electrochemical device that uses a solid oxide as an electrolyte to split water into hydrogen and oxygen at high temperatures, typically above 500 °C. Besides, AEMs are fuel cells that utilize AEMs to transport hydroxide ions (OH^−)^ from the cathode to the anode. They combine hydrogen and oxygen to produce electricity, with water and heat as products [18,37,40].

While economic analysis studies of water electrolysis technologies like Alkaline and PEM have been extensively conducted due to their technical maturity, there has been a relatively lesser focus on SOEC and still it is in laboratory phase. Further research is necessary to assess and enhance the economic feasibility of SOEC based hydrogen production. Table 2 shows specific cost, efficiencies, operating conditions and lifetime of several categories of electroylzer.

**Table 2:** Cost estimates and operating parameters for several types of electrolyzer.

Electrolyzer	Specific cost ($/kW)	System efficiency (kWh/kgH_2)_	Operating conditions	Lifetime (hours)	Present scenerio	Reference
Alkaline	860–1240	50–78	H_2_ produced at 30 bar, 80 °C	60000	Commercialize	[18,22]
PEM	1350–2200	50–83	H_2_ produced at less than 30 bar, 80 °C	50000–80000	Commercialize	[37,44]
AEM	400–800	57–69	H_2_ produced at less than 30 bar, 80 °C	>5000	Lab scale	[40]
SOEC	>1045	45–55	H_2_ produce at 30 bar, 700–800 °C	<20000	Lab scale	[40]

To accurately predict the levelized cost of hydrogen (LCOH) in 2030, 2040, and 2050, it is essential to have knowledge of the capital costs of the individual components involved. As the world continues to witness a surge in the adoption of hydrogen as an energy source, various sovereign and corporate climate change commitments are expected to result in a higher proportion of hydrogen in the global energy mix over the next decade and beyond, up until 2050. Table 3 shows the predicted cost of power producing technology using several renewable energy sources. Thermal energy storage (TES) technology has a vital function in concentrated solar power (CSP) plants which has capacity for storing heated water and can produce electricity during night and no sunshine for certain period of time.

**Table 3:** Forecasted cost of several green energy technologies [35,47].

Renewable Sources	CAPEX ($/kW)		
	2030	2040	2050
**Solar energy**			
CSP (without TES)	3760	3085	2780
PV systems (Without battery storage)	635	560	490
PV systems (With battery storage)	827	710	590
**Wind Turbine**			
Wind Turbine (Offshore)	2075	1730	1545
Wind Turbine (Onshore)	765	620	535
**Hydroelectric plant**	2401	2185	2180
**Geothermal**	4780	4525	4350

A sensitivity analysis is conducted for the year 2023 to identify the influencing parameters on net present value (NPV) for the current study, including capital expenditure (CAPEX), operating expenditure (OPEX), discount rate, cost of electricity production, and capacity factor of the hybrid systems. The study presents different scenarios for hybrid hydrogen production and analyzes the change in NPV when the cost of a specific component increases or decreases by 20% from the reference value of NPV, with other variables held constant.

## 4 Results and discussion

### 4.1 Minimum cost of hydrogen production

The presented Table 4 displays different renewable energy sources and their corresponding annual production capacity, annual equivalent cost, operational expenses, and capacity factor. To determine the minimum cost of hydrogen production, it is crucial to have knowledge of the annual equivalent cost, which can be obtained by knowing the annual production of each system, derived from their capacity factors. The operational expenses are calculated by considering 2% of the capital expenditure (CAPEX). This study utilizes the terms “Low Capacity Factor” (LCF) to represent the minimum and “High Capacity Factor” (HCF) to represent the maximum, for the purpose of estimating the cost of hydrogen production. It is also assumed that to produce 1 kg of hydrogen, 50 kWh of electric energy is needed [22].

**Table 4:** Estimation of annual equivalent cost for various renewable technologies.

Renewable Sources	Annual production (kWh)-LCF	Annual production (kWh)-HCF	Annual Equivalent Cost ($)	OPEX (Annually)	Capacity factor
Solar energy					
CSP (without TES)	2628	4380	459.48	129.52	30–50%
PV systems (Without battery storage)	876	2190	62.65	17.66	10–25%
PV systems (With battery storage)	1752	4380	85.63	24.14	20–50%
**Wind Turbine**					
Wind Turbine (Offshore)	3504	5256	263.94	74.4	40–60%
Wind Turbine (Onshore)	2190	3942	97.20	27.4	25–45%
**Hydroelectric plant**	2628	4380	164.45	50.56	30–50%
**Geothermal plant**	6132	7884	470.34	132.58	70–90%
Electrolyzer (Alkaline)	2628	4380	65.98	18.6	30–50%
Compressor			240.87	37.2	
ORC system	700.8	963.6	248.33	70	8–11%
Hydrogen tank ($/kg)			24.83	7	

**Fig. 2:**
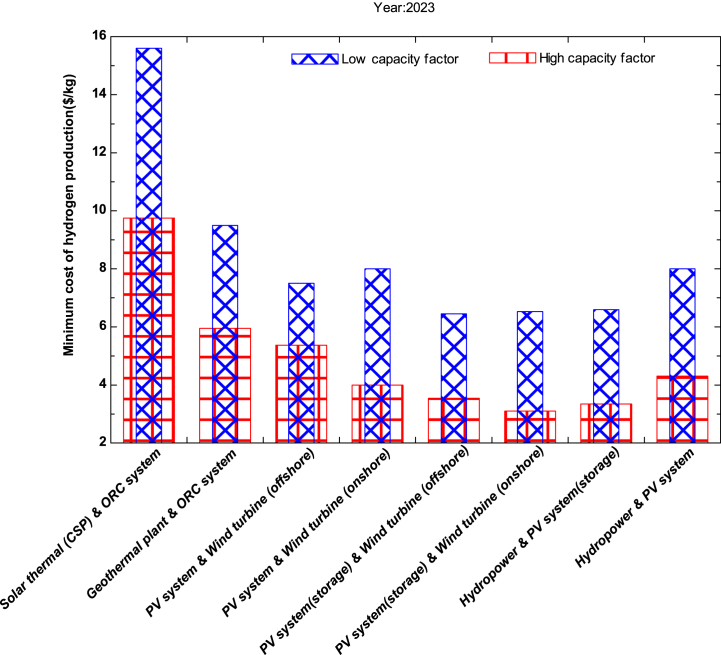
Minimum cost of hydrogen production for the year 2023.

In Fig. 2, the hybrid systems solar thermal (CSP) and ORC system have the highest cost of hydrogen production among other hybrid systems, being $15.6 and $9.75 per kg when operated at low and high capacity factors, respectively for the year 2023. The findings align with the research conducted by Gabreil et al. [48] and Sushant et al. [49], revealing that the cost of hydrogen using a solar Stirling-dish system was determined to be $10.49/kg and $10.68/kg respectively. Notably, the lowest production cost is observed in Saudi Arabia.

These two hybrid systems have higher capital expenditures in the current market due to the complexity and scale of the systems required for CSP technology, the need for large land area for minors to concentrate sunlight, the upfront investment in materials and installation, and the specialized components used. The integrated ORC plant also contributes to the higher cost of hydrogen due to the use of organic working fluids that are more expensive than water used in traditional steam turbines. Additionally, smaller ORC systems are more expensive per kW capacity than larger systems, and lower efficiency of these systems contributes to the higher cost of hydrogen production. On the other hand, the hybrid system consisting of solar PV (storage) and wind turbine (onshore) has the lowest cost of hydrogen production at $6.53 and $3.1 per kg for low and high capacity factors, respectively. If these systems are operated at higher capacity, the present cost may become more feasible in today's scenario.

**Fig. 3:**
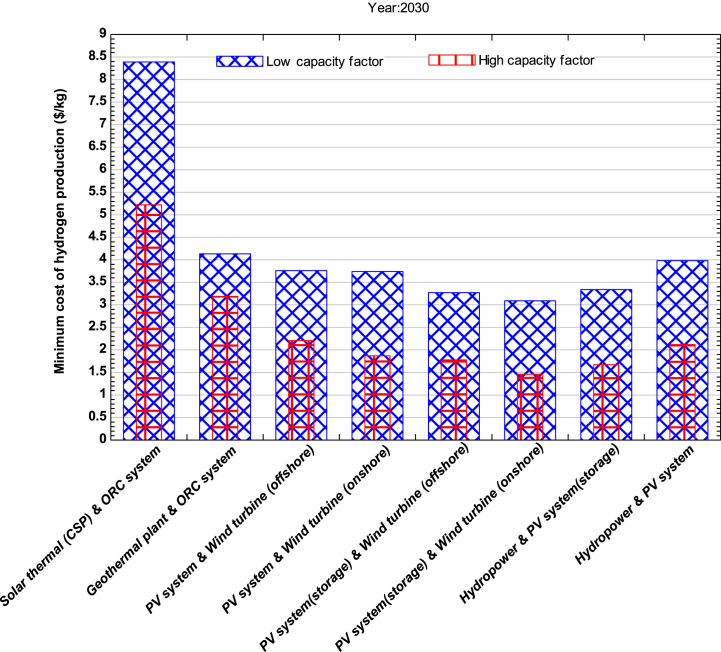
Minimum cost of hydrogen production for the year 2030.

Fig. 3 illustrates the estimated minimum cost of producing hydrogen in the year 2030. It is expected that the hybrid system comprising solar PV and onshore wind turbine will have the lowest cost, ranging between $1.46-$3.09 per kg of hydrogen. High capacity factor systems, particularly in regions with high solar insolation and wind velocity, will play a significant role in the production of hydrogen with minimum cost. Furthermore, it is anticipated that the cost of components such as PV modules, batteries, and power electronics will decrease, according to the report published by IRENA [3], resulting in a decrease in the LCOE of solar PV and wind energy by 59% and 18%, respectively. This will lead to the deployment of larger wind turbines with higher capacity factors, thereby lowering the LCOE. Countries such as Ireland, Germany, UK, Spain, Greece, Denmark, Uruguay, and Portugal have already installed solar PV and wind turbine in massive scale [50]. So such countries are expected to be global leaders in the production and export of hydrogen with minimum cost in 2030. Additionally, in the future, another emerging hydrogen production technology could be the hybrid system comprising solar PV without storage and offshore wind turbine, with a minimum cost between $1.78-$2.21 per kg of hydrogen. China, USA and South Korea are expected to be the highest hydrogen producing countries using a combination of onshore and offshore wind with solar PV hybrid systems. It is seen that the production cost of green hydrogen is high today and may remain high without subsidies or other supportive policies. Promising nations with potential for green hydrogen adoption lie within industries that manufacture steel and ammonia. The cost with satisfying electricity, heating, cooling and hydrogen needs can be met through the utilization of wind, water and solar technologies [51]. Therefore the presented results can have importance in the industries during the year 2030.

**Fig. 4:**
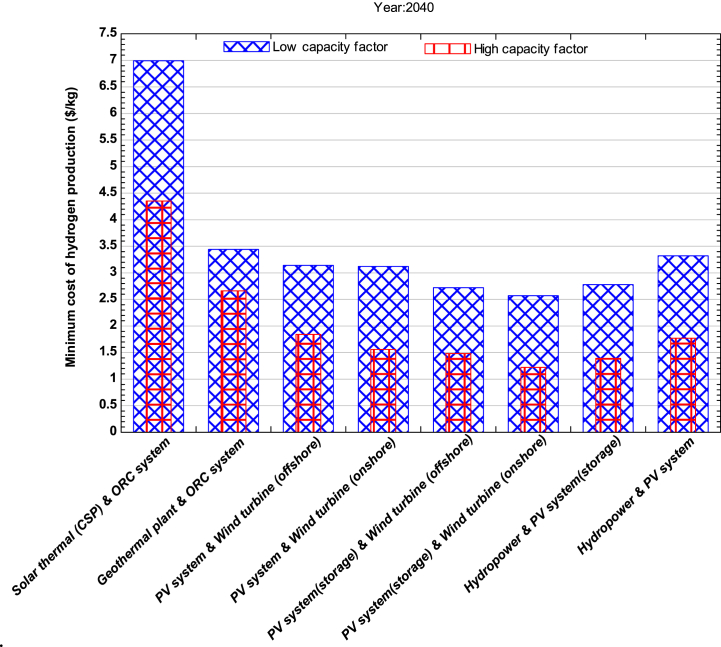
Minimum cost of hydrogen production for the year 2040.

Fig. 4 illustrates another potential hybrid system for hydrogen production in 2040, which comprises hydropower and solar PV with storage. Although hydropower is already a low-cost electricity production method, significant improvements in technology, including more efficient turbines, better control systems, and generators, could still be achieved. Furthermore, sedimentation issues could be reduced due to increased awareness and mitigation of climate change. With increased automation, lower construction costs, and improved energy storage, the cost of generation could also decrease. By 2040, the minimum cost of hydrogen production could range from $2.39 and $1.39 per kg for lower and higher capacity factor respectively. This hybrid system comprising of hydropower and solar PV system without storage could be economically feasible for systems with lower capacity factors. The cost of hydrogen production is expected to be between $2.27-$3.6 per kg. Countries like Sweden, Switzerland, Vietnam, Spain, Italy, France, Norway, Japan, and India, which have already installed large hydropower plants [52]. These countries could benefit from producing low-cost hydrogen by integrating it with a solar PV system.

Similarly, the research undertaken by Ref. [53] employs a geospatial modeling approach to ascertain the most economical means of producing green hydrogen across diverse low and middle-income nations. This innovative technique yields a cost to be projected to fall within the €1.8–3/kg by 2030. Even this modeling technique could forecast the cost of green hydrogen similar to our techno-economic analysis.

**Fig. 5:**
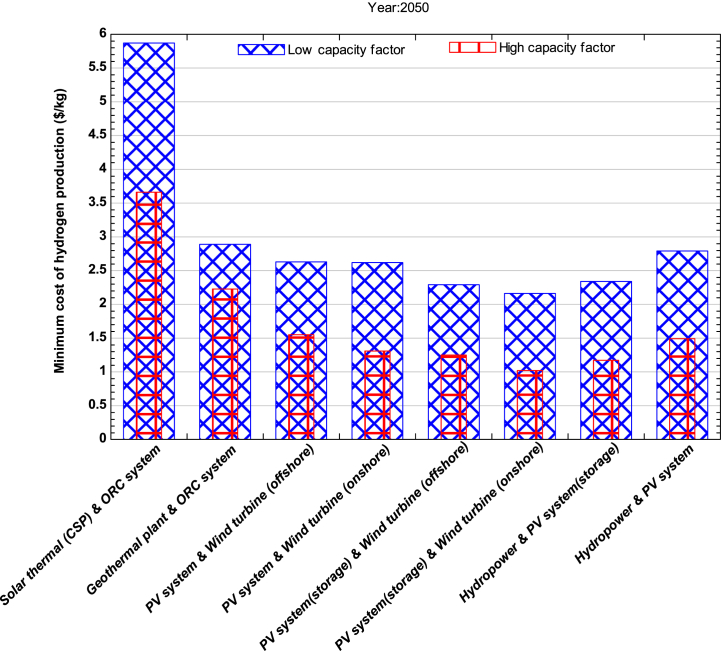
Minimum cost of hydrogen production for the year 2050.

The hybrid system depicted in Fig. 5 combines geothermal and solar thermal (CSP) systems with an ORC system and could be a promising technology for producing hydrogen, as well as power, fresh water, heating and cooling of buildings in the year 2050. The minimum cost of hydrogen production using this system is estimated to be in the range of $3.66-$5.87 per kg and $2.23-$2.89 per kg for solar thermal (CSP)and geothermal plant integrated with ORC system, respectively, when operating at high capacity factor. By 2050, the cost of drilling techniques may be reduced by up to 25%. Moreover, the discovery of new geothermal resources could significantly reduce the cost of producing electricity and, in turn, lower the cost of hydrogen production. Despite the cost of production being higher than other renewable sources, the efficiency of the ORC system could be increased by using organic fluids like iso-butane-R114 & R12-R114 with higher thermal efficiency of around 24–34% as reported [45]. This increase in efficiency could lower the cost of hydrogen production. On other hand, countries that have industries utilizing ORC systems for electrical power generation can reduce the cost of hydrogen. In particular, industries such as ceramic, cement, metal, and glass can generate hydrogen if the ORC system is solely responsible for producing electricity. The deployment of heat-to-power solutions can benefit from the low feed-in-tariffs price in the EU. Siloxane based ORCs, when used for low waste heat recovery, could be a viable option for producing low-cost hydrogen in both geothermal and solar thermal ORC systems [54,55]. The current ORC system is only cost-effective when used in large sizes for heat recovery [[56], [57]]. However, countries like Germany, Spain, and Italy can generate hydrogen at a lower cost. Another option for producing hydrogen is by using a matured electrolyzer such as SOEC, which is expected to be the best category for this type of hybrid system in 2050. Studies suggest that a 5% improvement in electrolyzer efficiency can decrease the LCOH by $0.8/kg for AE and $0.48 to $0.75/kg for PEM [3]. By the year 2050, other hybrid systems such as solar PV and wind, hydropower, and solar PV systems with or without storage will also be able to produce hydrogen at a minimum cost ranging between $1.02 to $1.55 per kg. The new method introduced by Ref. [58] could reduce GHG emissions from the use of green hydrogen that can be employed in steel mills for the countries such as China, India, Japan, US, Russia, South Korea and Germany and this option could be competitive by the year 2040. Based on this finding, the present study can have significant impact for the cheaper production of green hydrogen from the steel mills when integrated with ORC systems.

### 4.2 Sensitivity analysis

**Fig. 6:**
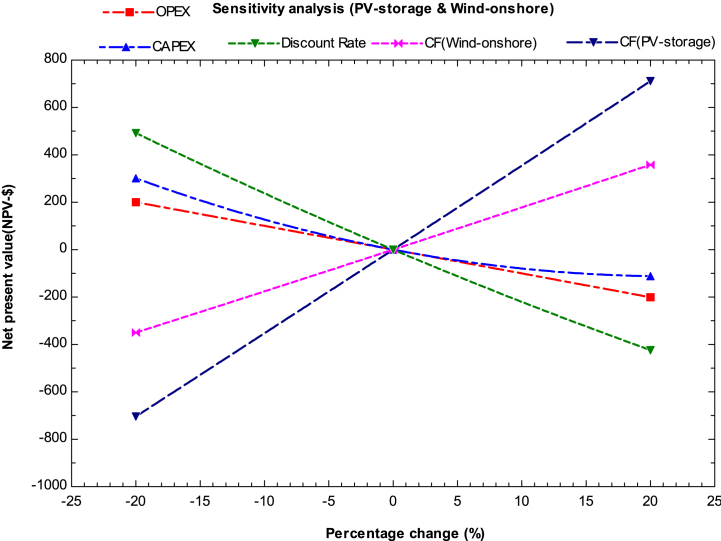
Sensitivity analysis for solar PV(storage) and wind onshore.

Fig. 6 illustrates the economic parameters for a hybrid system consisting of solar PV (with storage) and onshore wind turbines for hydrogen production, using a high capacity factor for analysis. The most sensitive cost component was found to be CAPEX, followed by the discount rate and capacity factor. The tentative payback period for this hybrid system ranges from 8 to 10 years, with an internal rate of return (IRR) range of 3.4–6.1% and a profitability index (PI) range of 1.18–1.41. The LCOH production cost was found to be $4.7/kg in 2023, with an IRR of 5.064% needed for profitability. The least influencing parameter in this case is for OPEX. The hybrid system provides economically favorable conditions for hydrogen production.

**Fig. 7:**
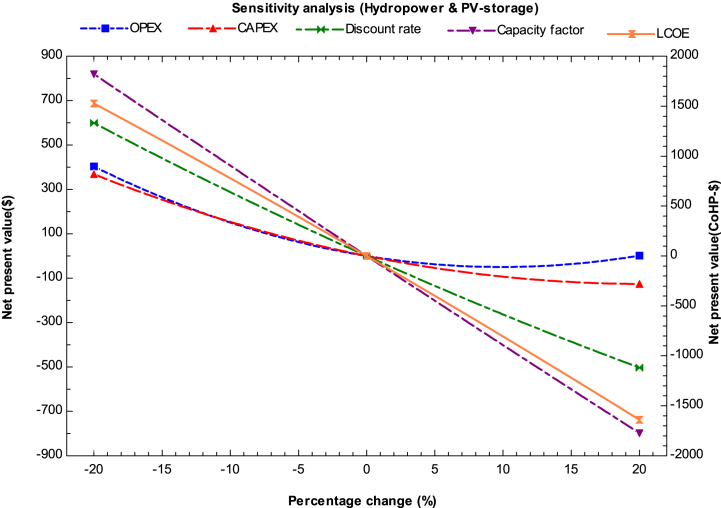
Sensitivity analysis for hydropower and solar PV (storage).

The sensitivity analysis for the hybrid system integrated with hydropower and solar PV (storage) is presented in Fig. 7. Both systems have the same capacity factor of 50%. The tentative payback period ranges from 9 to 11 years, while the IRR ranges from 1.87 to 7.74% and PI ranges from 1.05 to 1.45. The cost of electricity production and capacity factor are the most influential factors, followed by CAPEX and discount rate. The LCOE is found to be $5.15/kg, and the optimal profit requires a payback period of 9 years with an IRR of 5.7%.

**Fig. 8:**
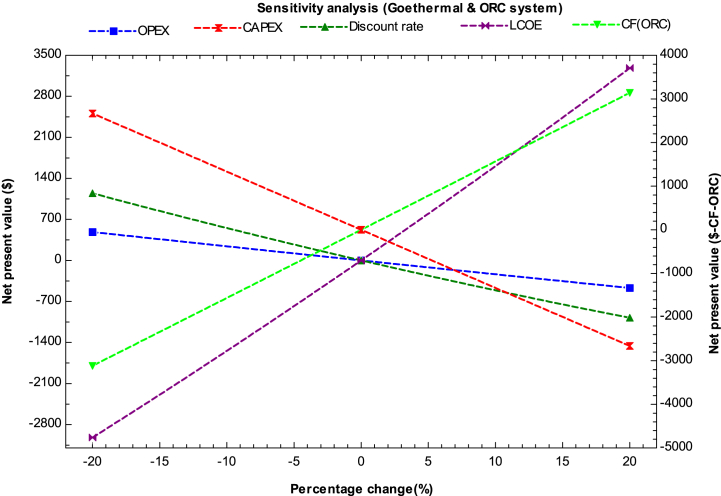
Sensitivity analysis for geothermal and ORC system.

Fig. 8 depicts the sensitivity analysis for a hybrid system composed of geothermal plant and ORC systems. The graph shows that the most significant factor influencing the change in NPV is the levelized cost of electricity (LCOE). CAPEX has the second highest impact on NPV, followed by capacity factor. It is observed that increasing the efficiency of the ORC plant can lead to a decrease in the cost of hydrogen. There is a slight impact on cost due to changes in the discount rate and OPEX. To be economically feasible, the LCOE production should be reduced to $9.09/kg with a payback period of 8 years and an IRR of 5.05%. During the sensitivity analysis, the IRR ranges from 2.017% to 8.04%, while the PI varies from 1.06 to 1.6. It is evident that geothermal and ORC systems have the potential to be a promising technology for hydrogen production if the efficiency of the ORC plant can be improved, leading to a reduction in the cost of hydrogen production.

**Fig. 9:**
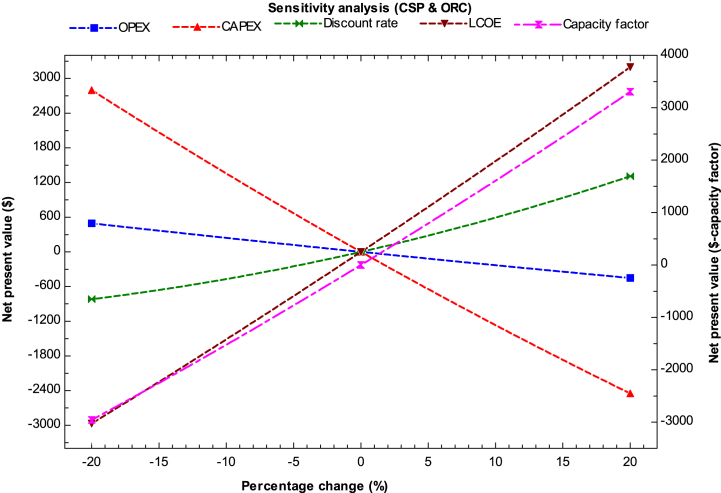
Sensitivity analysis for solar thermal (CSP) and ORC system.

Fig. 9 depicts the sensitivity analysis for a hybrid system consisting of solar thermal (CSP) and ORC systems to estimate the cost of hydrogen production. The graph reveals that the cost of hydrogen production is mainly affected by CAPEX, followed by capacity factor, LCOE, and discount rate. OPEX has a minimal effect on the cost. The IRR for this system ranges from 2.03 to 8.24%, while PI ranges from 1.06 to 1.6. The payback period in this case ranges from 8 to 12 years. In order to make this system economically viable, the LCOH production should be around $9.5/kg. The cost can be reduced for this hybrid system when the ORC systems operate throughout the year with more auxiliary power plants. Furthermore, the cost can be reduced if efficiency is increased. Currently, even if CAPEX is decreased, the cost of hydrogen production is still high. Recuperators indeed play an important role in maximizing the efficiency of an Organic Rankine Cycle (ORC) system for waste heat recovery. The use of a recuperator in the ORC system helps in improving its overall thermal efficiency and enhances sustainability [52]. However, in the long run, this system can become feasible when used for multi-generation purposes such as heating, cooling, and power generation from the hybrid unit.

## 5 Conclusions

The study examines the feasibility of green hydrogen production through different renewable hybrid and ORC systems. Eight scenarios are considered for hybrid systems, including solar PV with and without battery storage, onshore and offshore wind turbines, hydropower, geothermal plants, and solar thermal (CSP) plants integrated with ORC systems. The cost of hydrogen production is determined by reviewing various government reports and research literature and using governing equations for economics. The minimum cost of hydrogen production and the electricity required to produce 1 kg of hydrogen are calculated to predict the LCOH for each scenario in different years. This study has important implications for countries and regions to create effective and sustainable hydrogen strategies. The present study of the findings provides practical guidance for policymakers to develop well-informed policies and form systems that encourage the use of green hydrogen technologies. Additionally, the study helps to predict the future adoption of green hydrogen as a primary fuel source and highlights its importance for shaping energy trends. By analyzing different integration scenarios for cost-effectiveness, this study contributes to foresee how green hydrogen can be integrated into other renewable energy systems. Moreover, the thorough economic performance and sensitivity analysis it helps decision-makers to make appropriate choices when planning and implementing green hydrogen projects.

The study's main findings are summarized below.

1. In 2023, the estimated minimum cost of producing hydrogen using a combination of solar PV (with storage) and onshore wind turbines with alkaline water electrolysis was $3.1 per kg. On the other hand, the maximum cost of hydrogen production in the same year was with solar thermal (CSP) and ORC plants, which was $15.6/kg and $9.75/kg for lower and higher capacity factors, respectively. However, all the analyzed combinations currently have high production costs and cannot compete with hydrogen derived from fossil fuels.
2. The most promising hybrid systems for hydrogen production by the end of 2030 were found to be those combining solar PV and wind plants, both in onshore and offshore conditions. Additionally, other hybrid systems, such as hydropower and solar PV with storage, were also found to be favorable for hydrogen production. The estimated minimum cost of producing hydrogen using integrated solar PV and onshore wind was found to be in the range of $1.46 to $3.09 per kg for higher and lower capacity factors, respectively. Similarly, wind turbines in offshore conditions, integrated without storage, were also found to be feasible with a minimum hydrogen production cost ranging from $1.78 to $2.21 per kg in the same year.
3. The hybrid system integrated with an ORC system was found to be favorable for hydrogen production by 2050 when the ORC plant is installed in industries such as steel, glass, and metal to utilize waste heat for hydrogen production. Moreover, ORC integrated hydrogen production was found to be economically feasible with an electrolyzer (SOEC) because it operates at higher temperatures. The minimum cost of producing hydrogen was found to be $3.5/kg with higher capacity for CSP integrated ORC system by 2050. Furthermore, by 2050, geothermal-based ORC systems for hydrogen production could become competitive with other renewable technologies for multi-generation applications such as heating and cooling purposes.
4. According to the sensitivity analysis results, the most influential parameters for all the hybrid systems were found to be the LCOE (levelized cost of electricity) and capacity factor of the plant. Thus, for investors and planners to achieve a nominal profit on their investment, they should install the plant with high capacity and in areas with abundant energy resources. Operating expenditures were found to be the least influential factor, followed by the discount rate.
5. The study concluded that the LCOH for the year 2023 was approximately $4.7/kg with an IRR (internal rate of return) of 5.064% and a payback period of 8 years for the solar PV (storage) and onshore wind hybrid system when utilized at a higher capacity factor. The study also indicated that currently, none of the hybrid systems are economically viable. However, by 2030, two hybrid systems, solar PV integrated with wind turbines and hydropower, could compete with fossil fuel.

### 5.1 Policy implications and future direction

An integrated policy approach is important to overcome initial challenges and expand the use of green hydrogen. First, every nation should develop national hydrogen strategies, set policy priorities, and create a reliable system to produce green hydrogen. The plan can be divided from 2023 to 2050. From 2023 to 2030, the focus should be on scaling up electrolyser capacity to produce green hydrogen and decarbonize existing hydrogen applications. The years 2030–2040 aim to significantly increase electrolyser capacity along with production through solar PV, wind power and hydropower sources with a possible grid infrastructure for transporting and storing hydrogen. Lastly, from the year 2040–2050, green hydrogen is expected to be produced from utilizing the ORC technology from the industrial sectors which could be more cost effective. The study suggests several policy implications including providing incentives for manufacturers and developers, developing infrastructure and promoting international collaboration. To further advance green hydrogen, future research should focus on technology improvements in electrolyzer, optimization strategies for producing green hydrogen through solar PV and wind power.

## Data availability statement

The authors will provide data upon request.

## CRediT authorship contribution statement

**Suresh Baral:** Writing – original draft. **Juraj Šebo:** Writing – review & editing, Supervision.

## Declaration of competing interest

The authors declare that they have no known competing financial interests or personal relationships that could have appeared to influence the work reported in this paper.
